# Size and Shape of Protein Molecules at the Nanometer Level Determined by Sedimentation, Gel Filtration, and Electron Microscopy

**DOI:** 10.1007/s12575-009-9008-x

**Published:** 2009-05-15

**Authors:** Harold P Erickson

**Affiliations:** 1 Department of Cell Biology, Duke University Medical Center, Durham, NC, 27710-3709, USA

**Keywords:** Protein shape, hydrodynamics, gel filtration, sedimentation, electron microscopy

## Abstract

An important part of characterizing any protein molecule is to determine its size and shape. Sedimentation and gel filtration are hydrodynamic techniques that can be used for this medium resolution structural analysis. This review collects a number of simple calculations that are useful for thinking about protein structure at the nanometer level. Readers are reminded that the Perrin equation is generally not a valid approach to determine the shape of proteins. Instead, a simple guideline is presented, based on the measured sedimentation coefficient and a calculated maximum *S*, to estimate if a protein is globular or elongated. It is recalled that a gel filtration column fractionates proteins on the basis of their Stokes radius, not molecular weight. The molecular weight can be determined by combining gradient sedimentation and gel filtration, techniques available in most biochemistry laboratories, as originally proposed by Siegel and Monte. Finally, rotary shadowing and negative stain electron microscopy are powerful techniques for resolving the size and shape of single protein molecules and complexes at the nanometer level. A combination of hydrodynamics and electron microscopy is especially powerful.

## 1. Introduction

Most proteins fold into globular domains. Protein folding is driven largely by the hydrophobic effect, which seeks to minimize contact of the polypeptide with solvent. Most proteins fold into globular domains, which have a minimal surface area. Peptides from 10 to 30 kDa typically fold into a single domain. Peptides larger than 50 kDa typically form two or more domains that are independently folded. However, some proteins are highly elongated, either as a string of small globular domains or stabilized by specialized structures such as coiled coils or the collagen triple helix. The ultimate structural understanding of a protein comes from an atomic-level structure obtained by X-ray crystallography or nuclear magnetic resonance. However, structural information at the nanometer level is frequently invaluable. Hydrodynamics, in particular sedimentation and gel filtration, can provide this structural information, and it becomes even more powerful when combined with electron microscopy (EM).

One guiding principle enormously simplifies the analysis of protein structure. The interior of protein subunits and domains consists of closely packed atoms [1]. There are no substantial holes and almost no water molecules in the protein interior. As a consequence of this, proteins are rigid structures, with a Young's modulus similar to that of Plexiglas [2]. Engineers sometimes categorize biology as the science of "soft wet materials". This is true of some hydrated gels, but proteins are better thought of as hard dry plastic. This is obviously important for all of biology, to have a rigid material with which to construct the machinery of life. A second consequence of the close packed interior of proteins is that all proteins have approximately the same density, about 1.37 g/cm^3^. For most of the following, we will use the partial specific volume, *v*_2_, which is the reciprocal of the density. *v*_2_ varies from 0.70 to 0.76 for different proteins, and there is a literature on calculating or determining the value experimentally. For the present discussion, we will ignore these variations and assume the average *v*_2_ = 0.73 cm^3^/g.

## 2. How Big Is a Protein Molecule?

Assuming this partial specific volume (*v*_2_ = 0.73 cm^3^/g), we can calculate the volume occupied by a protein of mass *M* in Dalton as follows.

(2.1) $$\begin{array}{c} V(nm^{3})=\frac{\left(0.73\;cm^{3}/g)\times (10^{21}nm^{3}/cm^{3}\right)}{6.023\times 10^{23}Da/g}\times M(Da)\\ =1.212\times 10^{-3}(nm^{3}/Da)\times M(Da). \end{array}$$

The inverse relationship is also frequently useful: *M* (Da) = 825 *V* (nm^3^).

What we really want is a physically intuitive parameter for the size of the protein. If we assume the protein has the simplest shape, a sphere, we can calculate its radius. We will refer to this as *R*_min_, because it is the minimal radius of a sphere that could contain the given mass of protein

(2.2) $$\begin{array}{c} R_{\min}={\left(3V/4\pi \right)}^{1/3}\\ =0.066M^{1/3}\text{ (for }M\text{ in Dalton, }R_{\text{min}}\text{ in nanometer)}\text{.} \end{array}$$

Some useful examples for proteins from 5,000 to 500,000 Da are given in Table 1.

**Table 1:** *R*_min_ for proteins of different mass

Protein *M* (kDa)	5	10	20	50	100	200	500
*R*_min_ (nm)	1.1	1.42	1.78	2.4	3.05	3.84	5.21

It is important to emphasize that this is the minimum radius of a smooth sphere that could contain the given mass of protein. Since proteins have an irregular surface, even ones that are approximately spherical will have an average radius larger than the minimum.

## 3. How Far Apart Are Molecules in Solution?

It is frequently useful to know the average volume of solution occupied by each molecule, or more directly, the average distance separating molecules in solution. This is a simple calculation based only on the molar concentration.

In a 1-M solution, there are 6 × 10^23^ molecules/l, = 0.6 molecules/nm^3^, or inverting, the volume per molecule is *V* = 1.66 nm^3^/molecule at 1 M. For a concentration *C*, the volume per molecule is *V =* 1.66/*C*.

We will take the cube root of the volume per molecule as an indication of the average separation.

(3.1) $$d=V^{1/3}=1.18/C^{1/3},$$

where *C* is in molar and *d* is in nanometer. Table 2 gives some typical values.

**Table 2:** Distance between molecules as function of concentration

Concentration	1 M	1 mM	1 μM	1 nM
Distance between molecules (nm)	1.18	11.8	118	1,180

Two interesting examples are hemoglobin and fibrinogen. Hemoglobin is 330 mg/ml in erythrocytes, making its concentration 0.005 M. The average separation of molecules (center to center) is 6.9 nm. The diameter of a single hemoglobin molecule is about 5 nm. These molecules are very concentrated, near the highest physiological concentration of any protein (the crystallins in lens cells can be at >50% protein by weight).

Fibrinogen is a large rod-shaped molecule that forms a fibrin blood clot when activated. It circulates in plasma at a concentration of around 2.5 g/l, about 9 μM. The fibrinogen molecules are therefore about 60 nm apart, comparable to the 46-nm length of the rod-shaped molecule.

## 4. The Sedimentation Coefficient and Frictional Ratio. Is the Protein Globular or Elongated?

Biochemists have long attempted to deduce the shape of a protein molecule from hydrodynamic parameters. There are two major hydrodynamic methods that are used to study protein molecules—sedimentation and diffusion (or gel filtration, which is the equivalent of measuring the diffusion coefficient).

**Figure 1:**
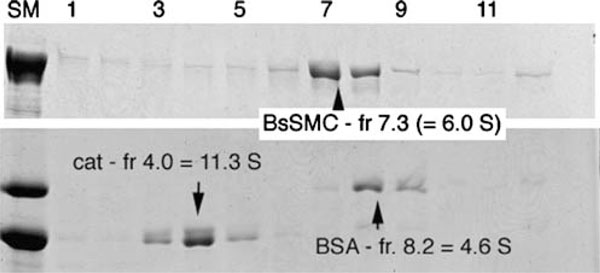
**Glycerol gradient sedimentation analysis of SMC protein from *B. subtilis* (*BsSMC*; *upper panel*) and sedimentation standards catalase and bovine serum albumin (*lower panel*)**. A 200-μl sample was layered on a 5.0-ml gradient of 15–40% glycerol in 0.2 M ammonium bicarbonate and centrifuged in a Beckman SW55.1 swinging bucket rotor, 16 h, 38,000 rpm, 20°C. Twelve fractions of 400 μl each were collected from a hole in the bottom of the tube and each fraction was run on SDS-PAGE. *Lane SM* shows the starting material, and fraction 1 is the bottom of the gradient. The *bottom panel* shows that the 11.3-S catalase eluted precisely in fraction 4, while the 4.6-S BSA eluted mostly in fraction 8, with some in fraction 9. We estimated the BSA to be centered on fraction 8.2. Experiments with additional standard proteins have demonstrated that the 15–40% glycerol gradients are linear over the range 3–20 S, so a linear interpolation is used to determine S of the unknown protein. BsSMC is in fractions 7 and 8, estimated more precisely at fraction 7.3. Extrapolating from the standards, we determine a sedimentation coefficient of 6.0 S for BsSMC. Other experiments gave an average value of 6.3 S for BsSMC [19].

The sedimentation coefficient, *S*, can be determined in an analytical ultracentrifuge. This was a standard part of the characterization of proteins in the 1940s and 1950s, and values of *S*_20,w_ (sedimentation coefficient standardized to 20°C in water) are collected in references such as the Chemical Rubber Co. (CRC) *Handbook of Biochemistry *[3]. Today, *S* is more frequently determined by zone sedimentation in a sucrose or glycerol gradient, by comparison to standard proteins of known *S*. Five to twenty percent sucrose gradients have been most frequently used, but we prefer 15–40% glycerol gradients in 0.2 M ammonium bicarbonate, because this is the buffer used for rotary shadowing EM (**Section 6**). The protein of interest is sedimented in one bucket of the swinging bucket rotor, and protein standards of known *S* (Table 5) are sedimented in a separate (or sometimes the same) gradient. Following sedimentation, the gradient is eluted into fractions and each fraction is analyzed by sodium dodecyl sulfate polyacrylamide gel electrophoresis (SDS-PAGE) to locate the standards and the test protein. Figure 1 shows an example determining the sedimentation coefficient of the structural maintenance of chromosome (SMC) protein from *Bacillus subtilis*.

The sedimentation coefficient of a protein is a measure of how fast it moves through the gradient. Increasing the mass of the protein will increase its sedimentation, while increasing its size or asymmetry will decrease its sedimentation. The relationship of *S* to size and shape of the protein is given by the Svedberg formula:

(4.1) $$S=M(1-v_{2}\rho )/N_{o}f=M(1-v_{2}\rho )/(N_{o}6\pi \eta R_{s}).$$

*M* is the mass of the protein molecule in Dalton; *N*_o_ is Avogadro's number, 6.023 × 10^23^; *v*_2_ is the partial specific volume of the protein; typical value is 0.73 cm^3^/g; *ρ* is the density of solvent (1.0 g/cm^3^ for H_2_O); *η* is the viscosity of the solvent (0.01 g/cm^-s^ for H_2_O).

A critical factor in the equation is the frictional coefficient, *f* (dimensions gram per second) which depends on both the size and shape of the protein. For a given mass of protein (or given volume), *f* will increase as the protein becomes elongated or asymmetrical (*f* can be replaced by an equivalent expression containing *R*_s_, the Stokes radius, to be discussed later). *S* has the dimensions of time (seconds). For typical protein molecules, *S* is in the range of 2–20 × 10^-13^ s, and the value 10^-13^ s is designated a Svedberg unit, S. Thus, typical proteins have sedimentation coefficients of 2–20 S.

From the above definition of parameters, it is clear that *S* depends on the solvent and temperature. In classical studies, the solvent-dependent factors were eliminated and the sedimentation coefficient was extrapolated to the value it would have at 20°C in water (for which *ρ* and *η* are given above). This is referred to as *S*_20,w_. In the present treatment, we will be referring mostly to standard proteins that have already been characterized, or unknown ones that will be referenced to these in gradient sedimentation, so our use of *S* will always mean *S*_20,w_.

A useful concept is the minimum value of *f*, which would obtain if the given mass of protein were packed into a smooth unhydrated sphere. As we have discussed in **Section 1**, the radius of this sphere will be *R*_min_ = 0.066 *M*^*1/3*^ (**Eq. 2.2**). In about 1850, G. G. Stokes calculated theoretically the frictional coefficient of a smooth sphere (note that the equation is similar to that for the Stokes radius, to be discussed later, but the parameters here are different):

(4.2) $$f_{\min}=6\pi \eta R_{\min}.$$

We have now designated *f*_min_ as the minimal frictional coefficient for a protein of a given mass, which would obtain if the protein were a smooth sphere of radius *R*_*min*_.

The actual *f* of a protein will always be larger than *f*_min_ because of two things. First, the shape of the protein normally deviates from spherical, to be ellipsoidal or elongated; closely related to this is the fact that the surface of the protein is not smooth but rather rough on the scale of the water molecules it is traveling through. Second, all proteins are surrounded by a shell of bound water, one–two molecules thick, which is partially immobilized or frozen by contact with the protein. This water of hydration increases the effective size of the protein and thus increases *f*.

### 4.1. The Perrin Equation Does Not Work for Proteins

If one could determine the amount of water of hydration and factor this out, there would be hope that the remaining excess of *f* over *f*_min_ could be interpreted in terms of shape. Algorithms have been devised for estimating the amount of bound water from the amino acid sequence, but these generally do not distinguish between buried residues, which have no bound water and surface residues which bind water. Some attempts have been made to base the estimate of bound water based on polar residues, which are mostly exposed on the surface. A 0.3-g H_2_O/g protein is a typical estimate, but in fact, this kind of guess is almost useless for analyzing *f*.

In the older days, when there was some confidence in these estimates of bound water, physical chemists calculated a value called *f*_o_, which was the frictional coefficient for a sphere that would contain the given protein, but enlarged by the estimated shell of water (other authors use *f*_o_ to designate what we term *f*_min _[3,4]; we recommend using *f*_min_ to avoid ambiguity). The measured *f* for proteins was almost always larger than *f*_o_, suggesting that the protein was asymmetrical or elongated. A very popular analysis was to model the protein as an ellipsoid of revolution and calculate the axial ratio from *f*/*f*_o_, using an equation first developed by Perrin. This approach is detailed in most classical texts of physical biochemistry. In fact, the Perrin analysis always overestimates the asymmetry of the proteins, typically by a factor of two to five. It should not be used for proteins.

The problem is illustrated by an early collaborative study of phosphofructokinase, in which the laboratory of James Lee did hydrodynamics and our laboratory did EM [5]. We found by EM that the tetrameric particles were approximately cylinders, 9 nm in diameter and 14 nm long. The shape was therefore like a rugby ball, with an axial ratio of 1.5 for a prolate ellipsoid of revolution. The Lee group measured the molecular weight and sedimentation coefficient, determined *f* and estimated water of hydration and *f*_o_. They then used the Perrin equation to calculate the axial ratio. The ratio was five, which would suggest that the protein had the shape of a hot dog. The EM structure (which was later confirmed by X-ray crystallography) shows that the Perrin equation overestimated the axial ratio by a factor of 3.

Teller et al. [6] summarized the situation: "Frequently the axial ratios resulting from such treatment are absurd in light of the present knowledge of protein structure." They explained that the major problem with the Perrin equation is that it treats the protein as a smooth ellipsoid, when in fact the surface of the protein is quite rough. Teller et al. went on to show how the frictional coefficient can actually be derived from the known atomic structure of the protein, by modeling the surface of the protein as a shell of small beads of radius 1.4 Å. The shell coated the surface of the protein, modeling its rugosity, and increasing the size of the protein by the equivalent of a single layer of bound water. This analysis has been extended by Garcia De La Torre and colleagues [7].

### 4.2. Interpreting Shape from ***f***/***f***_min_ = ***S***_max_/***S***

If the Perrin equation is useless, is there some other way that shape can be interpreted from *f*? The answer is yes, at a semiquantitative level. We have discovered simple guidelines where the ratio *f*/*f*_min_ can provide a good indication of whether a protein is globular, somewhat elongated, or very elongated.

Instead of proceeding with the classical ratio *f*/*f*_min_, where *f* is in nonintuitive units, we will reformulate the analysis directly in terms of the sedimentation coefficient, which is the parameter actually measured. We will define a value *S*_max_ as the maximum possible sedimentation coefficient, corresponding to *f*_min_*. S*_max_ is the *S* value that would be obtained if the protein were a smooth sphere with no bound water. These two ratios are equal: *f*/*f*_min_ = *S*_max_*/S*. Combining **Eqs. 2.2**, **4.1**, and **4.2**, we have

(4.3a) $$\begin{array}{c} S_{\max}=10^{13}M\;(1-v_{2}\rho )/N_{o}(6\pi \eta R_{\min})\\ =M\;[2.378\times 10^{-4}]/R_{\min} \end{array}$$

(4.3b) $$S_{\max}=0.00361M^{2/3}.$$

The leading factor of 10^13^ in **Eq. 4.3a** converts *S*_max_ to Svedberg units. The numbers in brackets in **Eq. 4.3a** are calculated using *v*_2_ = 0.73 cm^3^/g, *ρ* = 1.0 g/cm^3^, *η* = 0.01 g cm^-1^ s^-1^ = 10^-9^ g nm^-1^ s^-1^. The final expression, **Eq. 4.3b** expresses *S*_max_ in Svedbergs for a protein of mass *M* in Daltons. Some typical numerical values of *S*_max_ for proteins from 10,000 to 1,000,000 Da are given in Table 3.

**Table 3:** *S*_max_ calculated for proteins of different mass

**Protein *M***_**r **_**(kDa)**	10	25	50	100	200	500	1,000
*S*_max_ Svedbergs	1.68	3.1	4.9	7.8	12.3	22.7	36.1

We have surveyed values of *S*_max_/*S* for a variety of proteins of known structure. Table 4 presents *S*_max_/*S* for a number of approximately globular proteins and for a range of elongated proteins, all of known dimensions. It turns out that *S*_max_/*S* is an excellent predictor of the degree of asymmetry of a protein. From this survey of known proteins, we can propose the following general principals.

• No protein has *S*_max_/*S* = *f*/*f*_min_ smaller than ~1.2.

• For approximately globular proteins:

*S*_max_/*S* is typically between 1.2 and 1.3.

• For moderately elongated proteins:

*S*_max_/*S* is in the range of 1.5 to 1.9.

• For highly elongated proteins (tropomyosin, fibrinogen, extended fibronectin):

*S*_max_/*S* is in the range of 2.0 to 3.0.

• For very long thread-like molecules like collagen, or huge extended molecules like the tenascin hexabrachion (not shown):

*S*_max_/*S* can range from 3–4 or more.

**Table 4:** *S*_max_*/S* values for representative globular and elongated proteins

Protein	Dimensions (nm)	Mass	***S***_**max**_	*S*	***S***_**max**_**/*S***
Globular protein standards dimensions are from pdb files					
Phosphofructokinase	14 × 9 × 9	345,400	17.77	12.2	1.46
Catalase	9.7 × 9.2 × 6.7	230,000	13.6	11.3	1.20
Serum albumin	7.5 × 6.5 × 4.0	66,400	5.9	4.6	1.29
Hemoglobin	6 × 5 × 5	64,000	5.78	4.4	1.32
Ovalbumin	7.0 × 3.6 × 3.0	43,000	4.43	3.5	1.27
FtsZ	4.8 × 4 × 3	40,300	4.26	3.4	1.25
Elongated protein standards—tenascin fragments [27,28]; heat repeat [29,30]					
TNfn1–5	14.7 × 1.7 × 2.8	50,400	4.94	3.0	1.65
TNfn1–8	24.6 × 1.7 × 2.8	78,900	6.64	3.6	1.85
TNfnALL	47.9 × 1.7 × 2.8	148,000	10.1	4.3	2.36
PR65/A HEAT repeat	17.2 × 3.5 × 2.0	60,000	5.53	3.6	1.54
Fibrinogen	46 × 3 × 6	390,000	19.3	7.9	2.44

Apart from indicating the shape of a protein, *S*_max_/*S* can often give valuable information about the oligomeric state, if one has some idea of the shape. For example, if one knows that the protein subunit is approximately globular (from EM for example), but finds *S*_max_/*S* = 2.1, this would suggest that the protein in solution is actually a dimer. On the other hand, if one thinks a protein is a dimer, but finds *S*_max_/*S* < 1.0 for the dimer mass, the protein is apparently sedimenting as a monomer.

The use of *S*_max_/*S* to estimate protein shape has been described briefly in [8].

## 5. The Kirkwood/Bloomfield Calculation

The understanding of how protein shape affects hydrodynamics is elegantly extended by an analysis originally developed by Kirkwood [9] and later extended by Bloomfield and Garcia De La Torres [10-12]. In its simplest application, it calculates the sedimentation coefficient of a rigid oligomeric protein composed of subunits of known *S* and known spacing relative to each other. In more complex applications, a protein of any complex shape can be modeled as a set of nonoverlapping spheres or beads. *See* Byron [13] for a comprehensive review of the principals and applications of hydrodynamic bead modeling of biological macromolecules.

**Figure 2:**
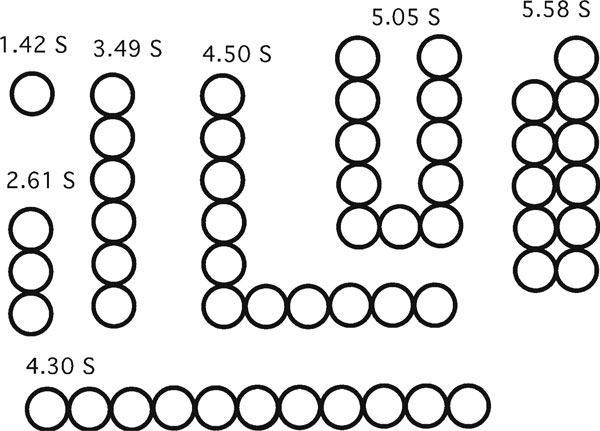
**Each bead models a 10-kDa domain, with an assumed sedimentation coefficient of 1.42 S**. The radius of the bead is 1.67 nm, using *R*_min_ = 1.42 nm, and adding 0.25 nm for a shell of water. The beads are an approximation to FN-III or Ig domains, which are ~1.7 × 2.8 × 3.5 nm. The sedimentation coefficients of multibead structures were calculated by the formula of Kirkwood/Bloomfield.

The basis of the Kirkwood/Bloomfield analysis is to account for how each bead shields the others from the effect of solvent flow and thereby determine the hydrodynamics of the ensemble from its component beads. Figure 2 shows a simple example of the bead modeling approach and provides an instructive look at how size and shape affect sedimentation. There are several important conclusions.

• A rod of three beads has about a twofold higher *S* than a single bead.

• *S*_max_/*S* is 1.18 for the single bead (the effect of the assumed shell of water), 1.34 for the three-bead rod, and 1.93 for the straight 11-bead rod. This is consistent with the principals given in **Section** 4 for globular, somewhat elongated, and very elongated particles.

• Bending the rod at 90° in the middle causes only a small increase in *S*. Bending it into a *U*-shape with the arms about one bead diameter apart increases *S* a bit more. Bending this same 11-bead structure more sharply, so the two arms are in contact, causes a substantial increase in *S*, from 5.05 to 5.58. The guiding principle is that folding affects *S* when one part of the molecule is brought close enough to another to shield it from water flow.

## 6. Gel Filtration Chromatography and the Stokes Radius

"Gel filtration chromatography is widely used for determining protein molecular weight." This quote from Sigma-Aldrich bulletin 891A is a widely held misconception. The fallacy is obscurely corrected by a later note in the bulletin that "Once a calibration curve is prepared, the elution volume for a protein of similar shape, but unknown weight, can be used to determine the MW." The key issue is "of similar shape". Generally, the calibration proteins are all globular, and if the unknown protein is also globular, the calibrated gel filtration column does give a good approximation of its molecular weight. The problem is that the shape of an unknown protein is generally unknown. If the unknown protein is elongated, it can easily elute at a position twice the molecular weight of a globular protein.

The gel filtration column actually separates proteins not on their molecular weight but on their frictional coefficient. Since the frictional coefficient, *f*, is not an intuitive parameter, it is usually replaced by the Stokes radius *R*_s_. *R*_s_ is defined as the radius of a smooth sphere that would have the actual *f* of the protein. This is much more intuitive since it allows one to imagine a real sphere approximately the size of the protein, or somewhat larger if the protein is elongated and has bound water.

As mentioned above for **Eq. 4.2**, Stokes calculated theoretically the frictional coefficient of a smooth sphere to be:

(6.1) $$f=6\pi \eta R_{\text{s}}.$$

The Stokes radius *R*_s_ is larger than *R*_min_ because it is the radius of a smooth sphere whose *f* would match the actual *f* of the protein. It accounts for both the asymmetry of the protein and the shell of bound water. More quantitatively, *f/f*_min_ = *S*_max_/*S* = *R*_s_/*R*_min_.

**Figure 3:**
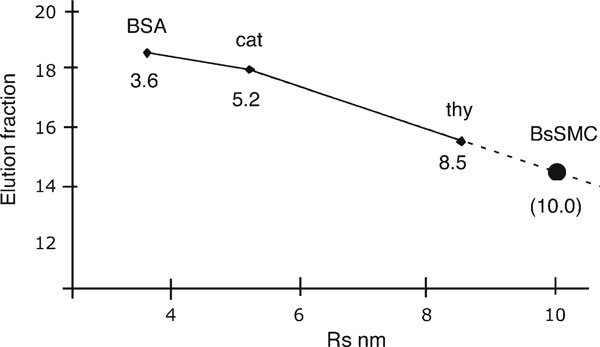
**Determination of *R*_s_ of BsSMC by gel filtration**. The column was calibrated by running standard proteins BSA, catalase, and thyroglobulin over the column, then BsSMC. BsSMC eluted in fraction 14.2, which corresponds to an *R*_s_ of 10 nm on the extrapolated curve. In repeated experiments, the average *R*_s_ was 10.3 nm [19].

Siegel and Monte [4] argued convincingly that the elution of proteins from a gel filtration column correlates closely with the Stokes radius, *R*_s_, presenting experimental data from a wide range of globular and elongated proteins. The Stokes radius is known for large number of proteins, including ones convenient for calibrating gel filtration columns (Table 5). Figure 3 shows an example where the *R*_s_ of the unknown protein SMC protein from *B. subtilis* was determined by gel filtration.

**Table 5:** Standards for hydrodynamic analysis

Protein	***M***_**r **_**aa seq**	***S***_**20,w**_	***S***_**max**_**/*S***	***R***_**s **_**(nm)**	Source	***M***_**r **_**S-M**
Ribonuclease A beef pancreas	14,044	2.0^a^	1.05^a^	1.64	HBC	13,791
Chymotrypsinogen A beef pancreas	25,665	2.6	1.21	2.09	HBC	22,849
Ovalbumin hen egg	42,910s	3.5	1.27	3.05	HBC	44,888
Albumin beef serum	69,322	4.6^a^	1.33	3.55	S-M, HBC	68,667
Aldolase rabbit muscle	157,368	7.3	1.45	4.81	HBC	147,650
Catalase beef liver	239,656	11.3	1.21	5.2	S-M	247,085
Apo-ferritin horse spleen	489,324	17.6	1.28	6.1	HBC	451,449
Thyroglobulin bovine	606,444	19	1.37	8.5	HBC	679,107
Fibrinogen, human	387,344	7.9	2.44	10.7	S-M	355,449

Gel filtration calibration kits, containing globular proteins of known molecular weight and *R*_s_, are commercially available (GE Healthcare, Sigma-Aldrich). These same proteins can be used for sedimentation standards. The proteins in these kits are included in the table along with some others that we have found useful. The values for *M*_r_ given in the first column are from amino acid sequence data. Values for *S*_20,*w*_ and *R*_s_ are from the Siegel–Monte paper (indicated S-M under source), or the CRC Handbook of Biochemistry [3] (indicated HBC). They agree with the values listed for *R*_s_ in the GE Healthcare gel filtration calibration kit, with the exception of ferritin. The "*M*_r_ calc" in the last column was obtained by our simplification of the Siegel–Monte calculation (*M* = 4,205 S *R*_s_). Note that the worst disagreement with "*M*_r_ aa seq" is about 10%

^a^*S* for ribonuclease A is questionable because of the low *S*_max_/*S* (1.05). *S* values for bovine serum albumin vary in the literature from 4.3 to 4.9. Many sources use 4.3, but we find that 4.6 gives a better fit with other standards (note that the standard curve in Figure 5 used 4.3, but 4.6 would have placed it closer to the line)

The standard proteins should span *R*_s_ values above and below that of the protein of interest (but in the case of SMC protein from *B. subtilis*, a short extrapolation to a larger value was used). The literature generally recommends determining the void and included volumes of the column and plotting a partition coefficient *K*_AV _[4]. However, we have found it generally satisfactory to simply plot elution position vs *R*_s_ for the standard proteins. This generally gives an approximately linear plot, but otherwise, it is satisfactory to draw lines between the points and read the *R*_s_ of the protein of interest from its elution position on this standard curve.

A gel filtration column can determine *R*_s_ relative to the *R*_s_ of the standard calibration proteins. The *R*_s_ of these standards was generally determined from experimentally measured diffusion coefficients. Some tabulations of hydrodynamic data list the diffusion coefficient, *D,* rather than *R*_s_, so it is worth knowing the relationship:

(6.2) $$D=kT/f=kT/(6\pi \eta R_{s}).$$

where *k* = 1.38 × 10^-16^ g cm^2^ s^-2^ K^-1^ is Boltzman's constant and *T* is the absolute temperature. *k* is given here in centimeter–gram–second units because *D* is typically expressed in centimeter–gram–second; *R*_*s*_ will be expressed in centimeter in this equation. Typical proteins have *D* in the range of 10^-6^ to 10^-7^ cm^2^ s^-1^. Converting to nanometer and for *T* = 300 K and *η* = 0.01:

(6.3) $$R_{s}=(1/D)\;2.2\times 10^{-6},$$

where *R*_s_ is in nanometer and *D* is in centimeter squared per second.

Simply knowing, *R*_s_ is not very valuable in itself, except for estimating the degree of asymmetry, but this would be the same analysis developed above for *S*_max_/*S*. However, if one determines both *R*_s_ and *S*, this permits a direct determination of molecular weight, which cannot be deduced from either one alone. This is described in the next section.

## 7. Determining the Molecular Weight of a Protein Molecule—Combining *S* and *R*_s_ à la Siegel and Monte

With the completion of multiple genomes and increasingly good annotation, the primary sequence of almost any protein can be found in the databases. The molecular weight of every protein subunit is therefore known from its sequence. But an experimental measure is still needed to determine if the native protein in solution is a monomer, dimer, or oligomer, or if it forms a complex with other proteins. If one has a purified protein, the molecular weight can be determined quite accurately by sedimentation equilibrium in the analytical ultracentrifuge. This technique has made a strong comeback with the introduction of the Beckman XL-A analytical ultracentrifuge. There are a number of good reviews [14,15], and the documentation and programs that come with the centrifuge are very instructive.

What if one does not have an XL-A centrifuge or the protein of interest is not purified? In 1966, Siegel and Monte [4] proposed a method that achieves the results of sedimentation equilibrium, with two enormous advantages. First, it requires only a preparative ultracentrifuge for sucrose or glycerol gradient sedimentation and a gel filtration column. This equipment is available in most biochemistry laboratories. Second, the protein of interest need not be purified; one needs only an activity or an antibody to locate it in the fractions. This is a very powerful technique and should be in the repertoire of every protein biochemist.

The methodology is very simple. The protein is run over a calibrated gel filtration column to determine *R*_s_ and hence *f*. Separately, the protein is centrifuged through a glycerol or sucrose gradient to determine *S*. One then uses the Svedberg equation (**Eq. 4.1**) to obtain *M* as a function of *R*_s_ and *S*.

(7.1a) $$M=SN_{o}(6\pi \eta R_{s})/(1-v_{2}\rho )$$

setting *η* = 0.01, *v*_2_*ρ* = 0.73, converting *S* to Svedberg units and *R*_*s*_ to nanometer, we can simplify further:

(7.1b) $$M=4,205\;(SR_{s})$$

where *S* is in Svedberg units, *R*_s_ is in nanometer, and *M* is in Daltons.

This is pretty simple! Importantly, in typical applications, this method gives the protein mass within about ±10%. This is more than enough precision to distinguish between monomer, dimer, or trimer.

**Table:** 

Application to SMC protein from *B. subtilis*. In the sections above, we showed how *S* of the SMC protein from *B. subtilis* was determined to be 6.3 S from glycerol gradient sedimentation, and *R*_*s*_ was 10.3 nm, from gel filtration. Putting these values in **Eq. 7.1b**, we find that the molecular weight of SMC protein from *B. subtilis* is 273,000 Da. From the amino acid sequence, we know that the molecular weight of one SMC protein from *B. subtilis* subunit is 135,000 Da. The Siegel–Monte analysis finds that the SMC protein from *B. subtilis* molecule is a dimer.
Knowing that SMC protein from *B. subtilis* is a dimer with molecular weight 270,000 Da, we can now determine its *S*_max_*/S*. *S*_max_ is 15.1 (**Eq. 4.3b**) so *S*_max_*/S* is 2.4. The SMC protein from *B. subtilis* molecule is thus expected to be highly elongated. EM (see below) confirmed this prediction.

## 8. Electron Microscopy of Protein Molecules

Since the early 1980s, electron microscopy has become a powerful technique for determining the size and shape of single protein molecules, especially ones larger than 100 kDa. Two techniques available in most EM laboratories, rotary shadowing and negative stain, can be used for imaging single molecules. Cryo-EM is becoming a powerful tool for protein structural analysis, but it requires special equipment and expertise. For a large number of applications, rotary shadowing and negative stain provide the essential structural information.

For rotary shadowing, a dilute solution of protein is sprayed on mica, the liquid is evaporated in a high vacuum, and platinum metal is evaporated onto the mica at a shallow angle. The mica is rotated during this process, so the platinum builds up on all sides of the protein molecules. The first EM images of single protein molecules were obtained by Hall and Slayter using rotary shadowing [16]. Their images of fibrinogen showed a distinctive trinodular rod. However, rotary shadowing fell into disfavor because the images were difficult to reproduce. Protein tended to aggregate and collect salt, rather than spread as single molecules. In 1976, James Pullman, a graduate student at the University of Chicago, then devised a protocol with one simple but crucial modification—he added 30% glycerol to the protein solution. For reasons that are still not understood, the glycerol greatly helps the spreading of the protein as single molecules.

Pullman never published his protocol, but two labs saw his mimeographed notes and tested out the effect of glycerol, as a part of their own attempts to improve rotary shadowing [17,18]. They obtained reproducible and compelling images of fibrinogen (the first since the original Hall and Slayter study and confirming the trinodular rod structure) and spectrin (the first ever images of this large protein). The technique has since been used in characterizing hundreds of protein molecules.

**Figure 4:**
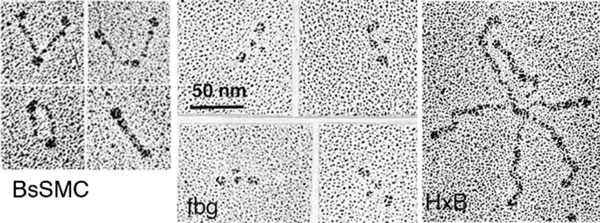
**Rotary shadowing EM of three highly elongated protein molecules: the SMC protein from *B. subtilis ***[19]**, fibrinogen **[20]**, and the hexabrachion protein, tenascin **[21].

Figure 4 shows rotary shadowed SMC protein from *B. subtilis*, fibrinogen, and hexabrachion (tenascin). SMC protein from *B. subtilis* is highly elongated, consistent with its high *S*_max_/*S* discussed above [19]. The fibrinogen molecules show the trinodular rod, but these images also resolved a small fourth nodule next to the central nodule [20], not seen in earlier studies. The central nodule is about 50 kDa, and the smaller fourth nodule is about 20 kDa. The "hexabrachion" tenascin molecule [21] illustrates the power of rotary shadowing at two extremes. First, the molecule is huge. Each of its six arms is made up of ~30 repeating small domains, totaling ~200,000 Da. At the larger scale, the EM shows that each arm is an extended structure, matching the length expected if the repeating domains are an extended string of beads. At the finer scale, the EM can distinguish the different sized domains. The inner segment of each arm is a string of 3.5-kDa epidermal growth factor domains, seen here as a thinner segment. A string of 10-kDa FN-III domains is clearly distinguished as a thicker outer segment. The terminal knob is a single 22-kDa fibrinogen domain. The *R*_min_ of these domains are 0.8, 1.7, and 2.8 nm, and these can be distinguished by rotary shadowing. Rotary shadowing EM can visualize single globular domains as small as 10 kDa (3.5 nm diameter) and elongated molecules as thin as 1.5 nm (collagen).

Negative stain is another EM technique capable of imaging single protein molecules. It is especially useful for imaging larger molecules with a complex internal structure, which appear only as a large blob in rotary shadowing. Importantly, noncovalent protein–protein bonds are sometimes disrupted in the rotary shadowing technique [8], but uranyl acetate, in addition to providing high resolution contrast, fixes oligomeric protein structures in a few milliseconds [22]. An excellent review of modern techniques of negative staining, with comparison to cryo-EM, is given in [23].

The simple picture of the molecule produced by EM is frequently the most straightforward and satisfying structural analysis at the 1–2-nm resolution. When the structure is confirmed by hydrodynamic analysis, the interpretation is even more compelling.

## 9. Hydrodynamic Analysis and EM Applied to Large Multisubunit Complexes

The text box above showed the application of the Siegel–Monte analysis to SMC protein from *B. subtilis*, which had only one type subunit and was found to be a dimer. Similar hydrodynamic analysis can be used to analyze multisubunit protein complexes. There are many examples in the literature; I will show here an elegant application to DASH/Dam1.

**Figure 5:**
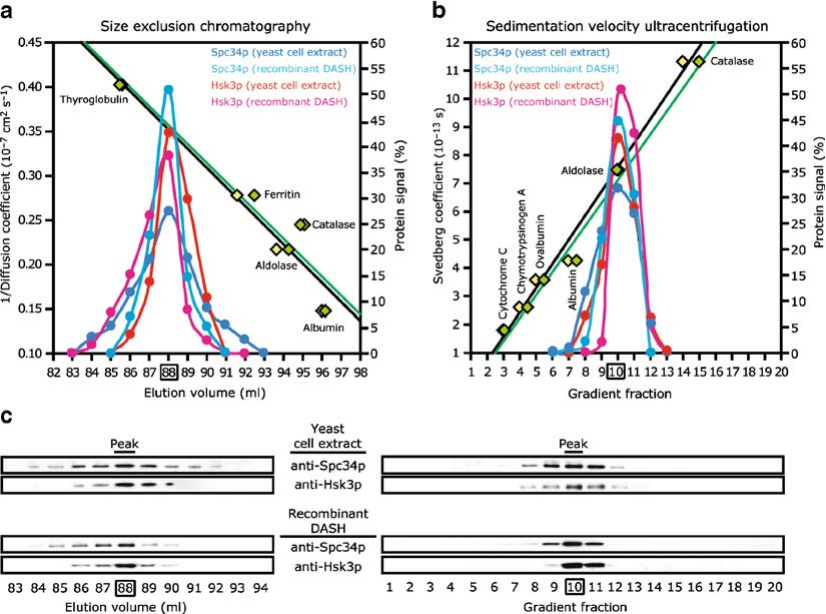
**Hydrodynamic analysis of the DASH/Dam1 complex**. Gel filtration is shown in **a** and sucrose gradient sedimentation in **b**. Independent calibration curves using standard proteins are shown in *black* and *green*. *Dark and light blue* show Spc34p in yeast cytoplasmic extract and in the purified recombinant protein. *Red and purple* show Hsk3p. The proteins were identified and quantitated by Western blot of the fractions, shown in **c**. The four protein bands eluted together at 1/*D* = 0.35 × 10^7^, corresponding to *R*_s_ = 7.6 nm, and at 7.4 S. Reproduced from Miranda et al. [24] with permission of the authors.

The protein complex called DASH or Dam1 is involved in attaching chromosomal kinetochores to microtubules in yeast. DASH/Dam1 is a complex of ten proteins that assemble into a particle containing one copy of each subunit. These complexes further assemble into rings that can form a sliding washer on the microtubule [24,25]. The basic ten-subunit complex has been purified from yeast and has also been expressed in *Escherichia coli* and purified (this required the heroic effort of expressing all ten proteins simultaneously [24]). Figure 5 shows the hydrodynamic characterization of the purified protein complex and illustrates several important features.

• For both the gel filtration (size exclusion chromatography, Figure 5a) and gradient sedimentation, Figure 5b, two calibration curves of known protein standards are shown, green and black. These are independent calibration runs. In this study, the gel filtration column was calibrated in terms of the reciprocal diffusion coefficient, 1/*D*, which is proportional to *R*_s_ (**Eq. 6.2**).

• The fractions were analyzed by Western blot for the location of two proteins of the complex, Spc34p and Hsk3p. Methods notes that 1 ml fractions from gel filtration were precipitated with perchloric acid and rinsed with acetone prior to SDS-PAGE, an essential amplification for the dilute samples of yeast cytoplasmic extract. These two proteins eluted together in both gel filtration and sedimentation, consistent with their being part of the same complex.

• The profiles of the two proteins were identical when analyzed in their native form in yeast cytoplasmic extract and as the purified complex expressed in *E. coli*. This is strong evidence that the expression protein is correctly folded and assembled.

• There is minimal trailing of any subunits. This means that there is no significant dissociation during the tens of minutes for the gel filtration, or the 12-h centrifugation. The complex is held together by very high affinity bonds, making it essentially irreversible.

• Combining the *R*_s_ = 7.6 nm (from 1/*D* = 0.35 × 10^-7^, and *S* = 7.4, **Eq. 7.1b** gives a mass of *M* = 236 kDa, close to the 204 kDa obtained from adding the mass of the ten subunits. *S*_max_ is 12.6 giving *S*_max_*/S* = 1.7, suggesting a moderately elongated protein.

**Figure 6:**
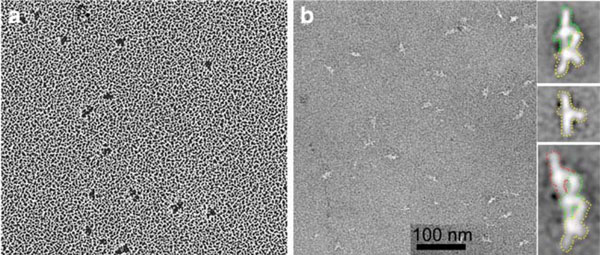
**EM of DASH/Dam1**. **a** Rotary shadowing shows particles roughly 13 nm in size, with irregular shape. **b** State-of-the-art negative stain coupled with single particle averaging shows a complex internal structure of the elongated particles. The scale bar indicates 100 nm for the unprocessed images. The averaged images on the *right* show a monomer, dimer, and trimer. These *panels* are 14 nm wide. The dimer was the predominant species. *Left panel* (rotary shadowing) reprinted with permission of Miranda et al. [24]. *Right panels* (negative stain) reprinted with permission of Wang et al. [26].

Figure 6 shows EM images of DASH/DAM1 by rotary shadowing (a) and negative stain (b). Rotary shadowing showed irregular particles about 13 nm in diameter [24]. The particles had variable and frequently elongated shapes, but internal structure could not be resolved. A later study used state of the art negative staining and sophisticated computer programs to sort images into classes and average them [26]. These images resolved a complex internal structure. The negative stain showed most of the particles (80%) to be dimers, with 15% monomers and 5% trimers. This contradicts the hydrodynamic analysis of Miranda et al. [24] showing that the particles were monomers. The reason for this discrepancy is not known.
